# Feeding Diqing Tibetan pigs with 50% of soybean meal replaced by walnut meal can reduce subcutaneous fat deposition and promote intramuscular fat accumulation

**DOI:** 10.3389/fmicb.2026.1794046

**Published:** 2026-03-19

**Authors:** Xinpeng Li, Siqi Jin, Hong Hu, Yi Lan, Bin Ni, Jianglong Su, Shuyuan Luo, Lei Tan, Yong Zhang, Hete Huang, Yin Xu, Jiayue Yang, Chuhuan Zhou, Keyan Chen, Shihao Li, Bingkun Liang, Siya Bai, Kang Zhang, Hongbin Pan, Xinxing Dong, Dawei Yan

**Affiliations:** Faculty of Animal Science and Technology, Yunnan Agricultural University, Kunming, Yunnan, China

**Keywords:** Diqing Tibetan pigs, integrated multi-omics analysis, intramuscular fat, soybean meal substitution, walnut meal

## Abstract

**Background:**

Protein feed resource shortage is a major constraint to the sustainable development of the livestock industry and a bottleneck problem hindering the growth of the Tibetan pig industry in China's Qinghai-Tibet Plateau region. Walnut meal, rich in protein, holds promise as a substitute for soybean meal. However, the effects and underlying mechanisms of walnut meal substitution on Tibetan pigs in Diqing remain unclear.

**Results:**

The study showed that substituting 50% of soybean meal with walnut meal in the diet of Diqing Tibetan pigs significantly reduced backfat thickness and increased intramuscular fat content (*P* < 0.05). Integrated multi-omics analyses, including metagenomics, transcriptomics, and lipidomics, revealed that walnut meal substitution significantly reduced the abundance of *Clostridium butyricum* in the cecum of Diqing Tibetan pigs. The reduction in *Clostridium butyricum* was linked to the lipolytic capacity of subcutaneous adipose tissue, potentially facilitating the breakdown of triglycerides into free fatty acids (FFAs), which are then released into the bloodstream. When these free fatty acids are transported to muscle tissue, the muscle exhibited inhibited oxidative metabolism (e.g., a decrease in acylcarnitine metabolites), while showing an upregulation in the expression of genes related to adipocyte differentiation (e.g., *MEDAG, VDR*) and triglyceride synthesis (e.g., *PPARGC*1*A, ANGPTL*4). Ultimately, these processes may contribute to the synthesis and storage of triglycerides in muscle, thereby facilitating intramuscular fat deposition.

**Conclusion:**

This study reveals that walnut meal can serve as a substitute for soybean meal, and a 50% substitution ratio is conducive to intramuscular fat deposition in Diqing Tibetan pigs. The findings provide valuable insights for the development and application of unconventional protein feed resources, and offer new perspectives for the production of marbled pork.

## 1 Introduction

Soybean meal is currently the most widely used plant protein raw material in the livestock industry. With increasing human demand for animal products and the rapid development of animal husbandry, substantial amounts of grain are being diverted to feed animals, leading to a prominent issue of “competition for grain between humans and livestock (Davis and D'Odorico, 2015).” To alleviate this conflict, many countries have begun emphasizing agricultural diversification and efficient resource utilization, actively exploring and developing alternative protein sources to reduce the overreliance on soybean meal in livestock farming (Moura et al., 2023). He et al. (2023) found that replacing soybean meal with mixed meals (rapeseed meal, cottonseed meal, and sunflower meal) had no significant impact on the average daily gain (ADG), average daily feed intake (ADFI), or feed-to-gain ratio of three-way crossbred pigs. GrabeŽ et al. (2020) discovered that using rapeseed meal and faba beans as alternative protein sources in pig diets not only caused no adverse effects on growth performance or carcass quality but also increased the content of free amino acids and sweet-tasting metabolites in pork.

The Diqing Tibetan pig is a type of Tibetan pig primarily distributed in Diqing Tibetan Autonomous Prefecture, Yunnan Province, China. It serves as a key breed for the swine industry in Diqing and other Tibetan-inhabited regions. However, due to Diqing's location on the southeastern edge of the Qinghai-Tibet Plateau, characterized by high-altitude, cold, and hypoxic conditions, as well as inconvenient transportation, high breeding costs have become a key bottleneck restricting the profitability of the Tibetan pig industry. Concurrently, walnuts are widely cultivated in Diqing's mountainous areas, and walnut meal—a by-product of walnut oil extraction—contains approximately 42% protein and 17% dietary fiber according to research, making it a valuable unconventional protein feed resource (Santos et al., 2018). Previous studies by our research team revealed that adding 5% walnut meal to the diet significantly upregulates the expression of lipid synthesis-related genes in adipose tissue of 3-month-old Diqing Tibetan pigs, suggesting a potential role in promoting fat deposition (Liu et al., 2024). Compared to early growth stages, finishing-stage Diqing Tibetan pigs exhibit higher nutritional demands, leading to increased consumption of protein feeds like soybean meal. Therefore, systematically evaluating the effects of appropriately increasing the substitution ratio of walnut meal for soybean meal during the finishing stage—particularly on growth performance and meat quality—and elucidating the regulatory mechanisms, could provide critical insights for the utilization of walnut meal in animal feed.

In this study, diets with different levels of walnut meal replacing soybean meal (0%, 25%, 50%, and 100%) were first formulated and fed to finishing Diqing Tibetan pigs, and the optimal replacement level was determined by comparing growth performance and carcass traits. Based on this, multiple tissues (muscle, liver, adipose tissue, and serum) as well as cecal contents of Diqing Tibetan pigs were subjected to multi-omics sequencing (transcriptomics, metabolomics, lipidomics, and metagenomics), and multi-omics integration was employed to investigate the potential molecular mechanisms by which walnut meal supplementation influences muscle growth through modulation of the gut microbiota.

## 2 Materials and methods

### 2.1 Diet formulation with walnut meal for Diqing Tibetan pigs

The conventional nutrient composition of walnut meal was first analyzed (Table 1). Diets were formulated based on the protein content of walnut meal. The control group (0W) was fed a corn-soybean meal-based diet, while the experimental groups (25W, 50W, and 100W) received diets in which 25%, 50%, and 100% of soybean meal were replaced with walnut meal, respectively. The diet formulations are presented in Table 2.

**Table 1:** Conventional nutrient composition of walnut meal.

**Crude protein (%)**	**Crude fat (%)**	**Crude fiber (%)**	**Crude ash (%)**	**Calcium (%)**	**Phosphorus (%)**	**Neutral detergent fiber (%)**	**Acid detergent fiber (%)**
22.62	8.17	29.37	2.78	0.45	0.52	49.26	33.14

**Table 2:** Formulation of experimental diets containing walnut meal.

**Items**	**Control (0W)**	**Experimental (25W)**	**Experimental (50W)**	**Experimental (100W)**
Corn	67.60	64.95	62.42	49.30
Soybean meal	12.00	9.00	6.00	0.00
Wheat bran	14.90	14.90	14.90	23.25
Soybean oil	3.00	1.67	0.30	0.00
Walnut meal	0	6.90	13.74	24.69
Lys	0	0.08	0.14	0.26
Premix	2.50	2.50	2.50	2.50
Total	100	100.00	100	100.00
Nutrient levels		
Crude protein	13.52	13.53	13.53	13.52
Digestible energy (MJ/kg)	13.85	13.86	13.81	13.85
Calcium	0.74	0.77	0.79	0.82
Available phosphorus	0.34	0.33	0.33	0.34
Lysine	0.94	0.95	0.94	0.94
Sodium chloride	0.02	0.02	0.02	0.02

### 2.2 Feeding trial

A total of 72 healthy, castrated, large-sized Diqing Tibetan pigs with similar genetic backgrounds, age (6 months), and body weight (approximately 70 kg) were randomly divided into four groups, with three pens per group and 6 pigs per pen as replicates. A pen-based design was adopted to ensure that all six pigs in each pen had sufficient space for feeding, avoiding large individual differences in feed intake caused by competition, which could potentially bias the experimental results. All pigs were raised under identical management conditions. The trial was supervised by dedicated personnel, with daily records of feed intake, remaining feed, and herd health. Feeding amounts were adjusted based on residual feed in the troughs, ensuring a small amount of leftover feed after each feeding. The pigs were fed three times daily at approximately 9:00, 14:00, and 20:00. The feeding trial lasted for 12 weeks.

### 2.3 Growth performance and slaughter measurements

At the beginning of the feeding trial, the initial body weight (IBW, kg) of each pig was measured using an electronic scale. During the trial, individual feed intake was recorded daily to calculate average daily feed intake (ADFI, kg). At the end of the feeding trial, the final body weight (FBW, kg) of each pig was measured, and average daily gain (ADG, g) was calculated based on the difference between the final and initial body weight divided by the number of feeding days.

At the end of the feeding trial, slaughter performance measurements were conducted. Feed was withheld from the 72 pigs scheduled for slaughter for 12 h before slaughter. All procedures complied with animal welfare standards and were approved by the Animal Ethics Committee of Yunnan Agricultural University (Approval No. 202401001).

Individual live weight was recorded before slaughter. After slaughter, carcass traits were evaluated according to standard procedures. Average backfat thickness, 6–7th intercostal backfat thickness, and skin thickness were measured using vernier calipers (mm). Carcass straight length and carcass oblique length were measured using a measuring tape (cm). The loin eye area was determined at the 6–7th rib section (cm^2^). Dressing percentage, lean meat percentage, skin ratio, bone ratio, and fat ratio were calculated based on carcass weight and the weights of separated tissues. The experimental data were analyzed using the general linear model (GLM) of SPSS 27.0 software. Multiple comparisons were performed using Duncan's test. Results are presented as “mean ± standard error (SE)”, with *P* < 0.05 indicating a significant difference and *P* < 0.01 indicating a highly significant difference.

### 2.4 Measurement of intramuscular fat content

Samples of the Longissimus thoracis muscle were collected from a total of 12 pigs [six from the control group (0W) and six from the 50W group] and immediately placed in a foam box containing pre-prepared frozen ice packs. These 12 pigs were also used for the corresponding omics analyses. The samples were transported to the laboratory under refrigerated conditions. Upon arrival, visible surface fat and connective tissue were removed. The samples were blotted with filter paper to remove surface moisture, rapidly frozen in liquid nitrogen, and ground into fine powder using a tissue grinder.

Approximately 2.0 g of muscle powder were accurately weighed and subjected to fat extraction using the Soxhlet method according to the national standard GB 5009.6-2016. Anhydrous ether was used as the solvent, and the samples were placed in extraction thimbles and refluxed in a 70 °C water bath. Each hour, 6–8 reflux cycles were performed, and the extraction was continued for 8 h. After extraction, the solvent was recovered and evaporated, and the extraction bottles were dried in a 105 °C oven to constant weight. The total mass of the extraction bottle and extracted fat was accurately measured. The intramuscular fat content was calculated by dividing the mass of extracted fat by the initial mass of muscle powder and expressed as a percentage.

The differences in intramuscular fat content between the 0W and 50W groups were analyzed using an independent-samples *t*-test, with *P* < 0.05 considered statistically significant.

### 2.5 Cecum content metagenomic sequencing analysis

At the time of slaughter, the abdominal cavity was quickly opened, and the cecum was located. Since the cecum harbors the richest microbial community in the pig gut, the middle section of the cecum was incised using a sterile surgical knife, and the cecum contents were collected into sterile tubes. The same 12 pigs (six per group) used for intramuscular fat measurement were used as biological replicates for metagenomic sequencing. The samples were immediately placed in liquid nitrogen and transported back to the laboratory for sequencing.

Genomic DNA was extracted from the cecum contents using the Tiangen Stool Genomic DNA Extraction Kit (DP328) according to the manufacturer's instructions. The quality of the extracted DNA was assessed by 1% agarose gel electrophoresis. Qualified samples were subjected to metagenomic sequencing on the Illumina NovaSeq 6000 platform using the PE150 strategy.

First, the raw sequencing data were filtered and quality-controlled using the fastp (Chen et al., 2018) software. Then, the clean data were aligned to the pig reference genome (version: Sscrofa11.1: https://ftp.ensembl.org/pub/release-115/fasta/sus_scrofa/dna/Sus_scrofa.Sscrofa11.1.dna.toplevel.fa.gz) using Bowtie2 (Jung and Han, 2022). Finally, sequences that did not align to the host genome were extracted using SAMtools (Danecek et al., 2021) and saved in FASTQ format.

The standard Kraken database (https://genome-idx.s3.amazonaws.com/kraken/k2_standard_20241228.tar.gz) was downloaded. This database contains reference sequences for archaea, bacteria, humans, vectors, and viruses. The Kraken (Lu et al., 2022) software was used to classify the sequences based on a k-mer alignment method, generating a taxonomic report. Subsequently, Bracken (Lu et al., 2017) was employed to correct the preliminary classification results and estimate relative abundance at the species level.

Principal Coordinate Analysis (PCoA) was performed using the vegan package in R to assess β-diversity, aiming to identify potential outlier samples and determine whether microbial community composition differed between groups.

To identify microorganisms with significantly different abundances between groups, Linear Discriminant Analysis (LDA) was conducted using the LEfSe (Segata et al., 2011) software. Microorganisms with an LDA score > 2 were considered significantly differentially abundant at the species level.

### 2.6 Transcriptome sequencing and bioinformatics analysis of back subcutaneous fat, liver, and longissimus thoracis muscle

After slaughter, liver, back subcutaneous fat, and Longissimus thoracis muscle samples were collected from the same 12 pigs (six from the 0W group and six from the 50W group) from which cecum contents had been collected. These tissues were selected because muscle growth is closely associated with fat deposition, and the liver plays a central role in lipid metabolism. All samples were immediately placed in sterile tubes pre-filled with RNA stabilization solution, flash-frozen in liquid nitrogen, and transported to the laboratory for transcriptome analysis.

Total RNA was extracted from the tissues using the Tiangen Animal Tissue Total RNA Extraction Kit (DP451) according to the manufacturer's instructions. The concentration and purity of the extracted RNA were first assessed using a spectrophotometer, and RNA integrity was then evaluated using the Agilent 2100 Bioanalyzer. Samples that passed quality control were subjected to transcriptome sequencing on the Illumina NovaSeq 6000 platform using the PE150 strategy.

First, the raw sequencing data (raw data) were filtered and quality-controlled using fastp (Chen et al., 2018). Then, the clean data were aligned to the pig reference genome (Sscrofa11.1) using HISAT2 (Kim et al., 2019). Finally, gene expression levels in each sample were quantified using the featureCounts (Liao et al., 2014) command from the Subread software package.

Differentially expressed genes (DEGs) between groups were identified using DESeq2 (Love et al., 2014), with the thresholds set at |log_2_(FoldChange)| > 1 and adjusted *P*-value (Padj) < 0.05.

### 2.7 Back subcutaneous fat, liver, longissimus thoracis muscle, and serum metabolome/lipidome sequencing and analysis

To assess whether changes in gene transcription levels affect the functional state of the tissues, we performed lipidomic analyses on back subcutaneous fat and liver, and metabolomic analyses on Longissimus thoracis muscle and serum using the same batch of tissue samples employed for transcriptome sequencing.

Blood samples were drawn from the marginal ear vein into anticoagulant-free tubes and allowed to clot naturally. After coagulation, the blood samples were centrifuged at 3,000 rpm for 10 min, and the upper layer of light yellow transparent serum was collected. All tissues and serum samples were placed in sterile tubes, immediately flash-frozen in liquid nitrogen, and transported to the laboratory for downstream analysis.

For lipidomic analysis of liver and fat, ultra-performance liquid chromatography-tandem mass spectrometry (UPLC-MS/MS) was performed based on the self-built Metware Metabolism lipid database, utilizing retention time (RT), parent-fragment ion pair information, and MS/MS data. Quantitative analysis was conducted in multiple reaction monitoring (MRM) mode using a triple quadrupole mass spectrometer.

For metabolomic analysis of muscle and serum, metabolites were similarly analyzed by UPLC-MS/MS. Multivariate statistical analysis was performed using Orthogonal Partial Least Squares-Discriminant Analysis (OPLS-DA). Differential metabolites were initially screened based on Variable Importance in Projection (VIP) values derived from the OPLS-DA model, and significantly differential metabolites were identified using the thresholds VIP > 1 and *P*-value < 0.05.

## 3 Results

### 3.1 Replacing 50% of soybean meal with walnut meal does not have a negative effect on the growth performance of Diqing Tibetan pigs

By comparing the growth performance of Diqing Tibetan pigs fed diets in which soybean meal was replaced with walnut meal at different levels (25%, 50%, and 100%) with those fed a complete feed, the results (Table 3) showed that the average daily gain (ADG) of the 50W group was the highest among all treatments, but the difference compared with the control group was not statistically significant (*P* > 0.05). Similarly, the final body weight of the 50W group was slightly higher than that of the control group, but the difference was also not significant (*P* > 0.05). These findings indicate that replacing 50% of soybean meal with walnut meal does not negatively affect the growth performance of Diqing Tibetan pigs.

**Table 3:** Effects of different walnut meal replacement ratios on the growth performance of Diqing Tibetan pigs.

**Item**	**Replacement ratio**			
	**0% (0W)**	**25% (25W)**	**50% (50W)**	**100% (100W)**
Initial body weight IBW/kg	69.19 ± 1.19	69.26 ± 0.99	70.08 ± 1.04	70.03 ± 1.18
Final body weight FBW/kg	130.60 ± 3.06^AB^	131.20 ± 4.03^AB^	134.41 ± 3.80^A^	127.57 ± 3.49^B^
Average daily gain ADG/g	731 ± 27^AB^	737 ± 50^AB^	765 ± 38^A^	685 ± 39^B^

Different lowercase letters above the same row indicate a significant difference (*P* < 0.05), different uppercase letters indicate a highly significant difference (*P* < 0.01), and the same letter or no letter indicates no significant difference (*P*>0.05).

### 3.2 Replacing 50% of soybean meal with walnut meal increases intramuscular fat content in Diqing Tibetan pigs

By comparing the carcass traits of Diqing Tibetan pigs fed diets in which 25%, 50%, or 100% of soybean meal was replaced with walnut meal to those fed a complete diet, the results showed that (Table 4) feeding 50% walnut meal (50W group) significantly reduced fat deposition. Specifically, the 50W group had significantly lower average backfat thickness (49.30 ± 4.06 mm) and 6–7th rib backfat thickness (55.67 ± 3.27 mm) compared with the control group 0W (60.41 ± 4.98 mm and 61.33 ± 3.95 mm, *P* < 0.05), and the fat ratio was also significantly decreased (33.39 ± 1.98% vs. 38.47 ± 1.92%, *P* < 0.05). These results indicate that replacing 50% of soybean meal with walnut meal can effectively reduce subcutaneous and overall fat deposition.

**Table 4:** Effects of different walnut meal replacement ratios on the carcass traits of Diqing Tibetan pigs.

**Item**	**Replacement ratio**			
	**0% (0W)**	**25% (25W)**	**50% (50W)**	**100% (100W)**
Live weight before slaughter/kg	130.60 ± 3.06	131.20 ± 4.03	134.41 ± 3.80	127.57 ± 3.49
Average backfat thickness/mm	60.41 ± 4.98^Aa^	53.90 ± 3.11^ABb^	49.30 ± 4.06^Bb^	49.90 ± 3.19^Bb^
6-7th rib backfat thickness/mm	61.33 ± 3.95^a^	60.65 ± 2.65^a^	55.67 ± 3.27^b^	56.67 ± 3.01^b^
Thick skin/mm	3.38 ± 0.44	3.36 ± 0.36	3.51 ± 0.45	3.74 ± 0.34
Carcass straight length/cm	92.10 ± 3.60	92.67 ± 4.52	94.00 ± 4.24	92.33 ± 3.53
Carcass oblique length/cm	75.67 ± 3.01	76.67 ± 2.31	76.50 ± 3.54	76.67 ± 2.08
Acreage of longissimus muscle/cm^2^	30.87 ± 3.13	31.46 ± 3.97	32.75 ± 4.06	29.61 ± 3.36
Dressing percentage/%	73.33 ± 1.84	71.91 ± 1.70	70.85 ± 2.01	71.64 ± 1.68
Lean meat percentage/%	46.27 ± 2.71^b^	47.70 ± 3.31^ab^	49.90 ± 2.22^a^	49.67 ± 2.71^ab^
Skin ratio/%	7.67 ± 1.03	7.44 ± 0.50	8.21 ± 0.15	8.07 ± 1.41
Bone ratio/%	7.62 ± 0.56	7.96 ± 1.44	8.49 ± 3.23	8.38 ± 0.39
Fat ratio/%	38.47 ± 1.92^Aa^	36.92 ± 2.69^ABa^	33.39 ± 1.98^Bb^	33.93 ± 2.62^Bb^

Different lowercase letters above the same row indicate a significant difference (*P* < 0.05), different uppercase letters indicate a highly significant difference (*P* < 0.01), and the same letter or no letter indicates no significant difference (*P*>0.05).

The reduction in fat deposition may affect meat quality. To investigate this, we measured intramuscular fat content in the muscles of the control group (0W) and the 50W group. The results showed that (Table 5), although subcutaneous fat deposition was reduced in the 50W group, intramuscular fat content was significantly higher than in the control group (5.14 ± 0.64% vs. 2.46 ± 0.37%, *P* < 0.05). These findings suggest that feeding 50% walnut meal may suppress subcutaneous fat accumulation while promoting intramuscular fat deposition, thereby influencing fat distribution and meat quality.

**Table 5:** Effects of 50% walnut meal replacement on intramuscular fat content of Diqing Tibetan pigs.

**Item**	**Replacement ratio**	
	**0% (0W)**	**50% (50W)**
Intramuscular fat content/%	2.46 ± 0.37^b^	5.14 ± 0.64^a^

Different lowercase letters above the same row indicate a significant difference (*P* < 0.05), different uppercase letters indicate a highly significant difference (*P* < 0.01), and the same letter or no letter indicates no significant difference (*P*>0.05).

### 3.3 A decrease in the abundance of *Clostridium butyricum* in the cecum is associated with enhanced lipolytic capacity of white adipose tissue and reduced backfat thickness

**Figure 1:**
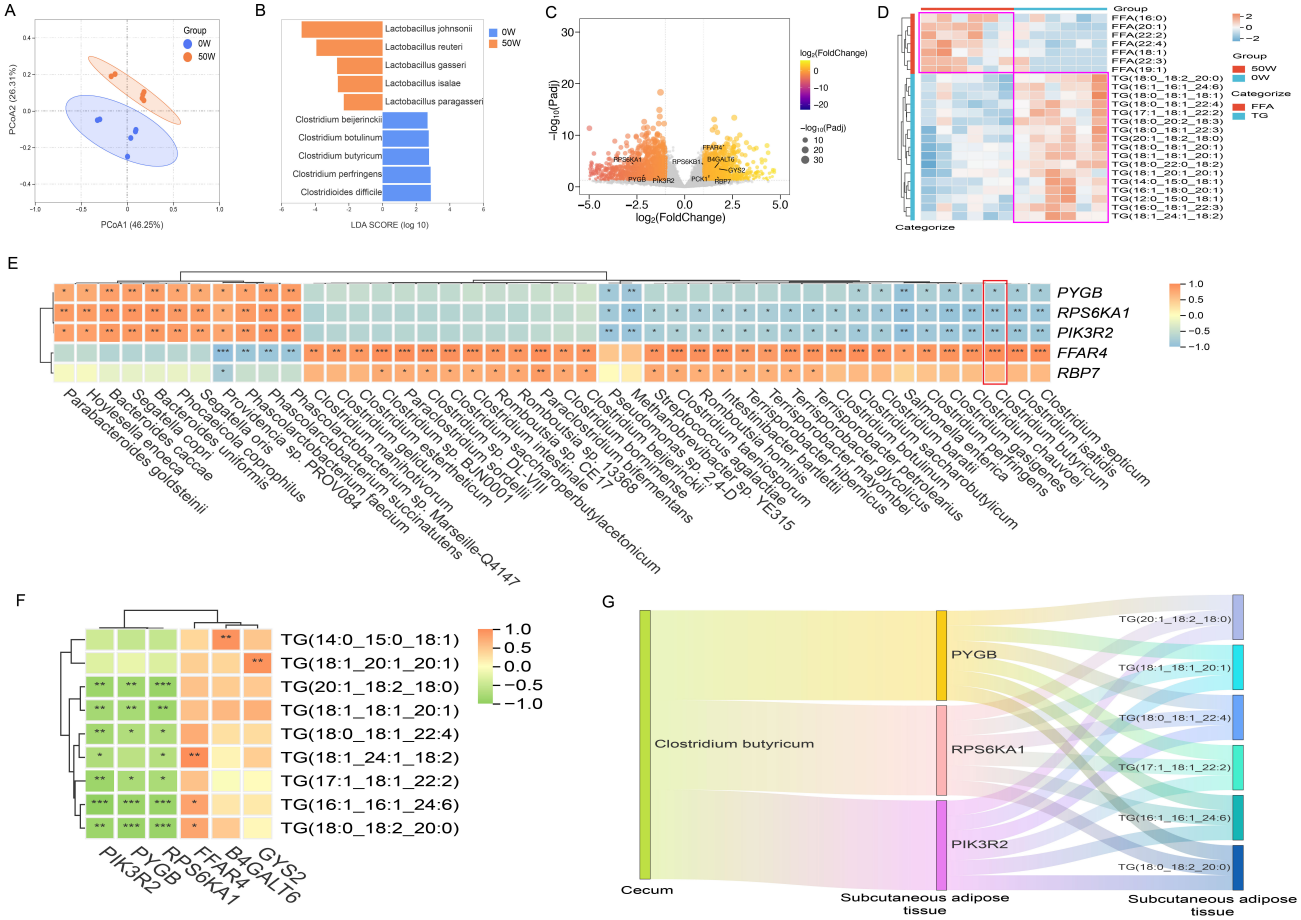
Integrated analysis of cecum content metagenome with transcriptome and lipidome of back adipose tissue. **(A)** PCoA analysis of the cecum content metagenome. **(B)** LEfSe analysis of the cecum content metagenome. **(C)** Transcriptomic analysis revealed differentially expressed genes in the subcutaneous adipose tissue between the 0W and 50W groups. **(D)** Lipidomic analysis revealed differences in triglyceride and free fatty acid metabolites in the subcutaneous adipose tissue between the 0W and 50W groups. **(E)** Correlation analysis between cecum metagenome and subcutaneous adipose tissue transcriptome. **(F)** Correlation analysis between subcutaneous adipose tissue transcriptome and lipidome. **(G)** Sankey diagram showing how changes in Clostridium butyricum abundance affect adipose gene expression and lipid metabolism, leading to triglyceride reduction and enhanced lipolysis.

In the species-level community composition and abundance analysis of cecum microbiota from the control group (0W) and experimental group (50W), PCoA analysis (Figure 1A) revealed clear separation between the two groups, indicating differences in β diversity. This suggests that replacing 50% soybean meal with walnut meal in the diet of Diqing Tibetan pigs altered their cecum microbial communities. LEfSe analysis based on grouping information showed that 84 microbial taxa at the species level exhibited significant differences in relative abundance between the 0W and 50W groups (LDA score > 2). Specifically, the abundance of *Lactobacillus reuteri* was significantly higher in the 50W group, while *Clostridium butyricum* was significantly lower (Figure 1B). Previous studies have demonstrated that dietary supplementation with *Lactobacillus reuteri* reduces backfat thickness in pigs (Cao et al., 2022), whereas *Clostridium butyricum* promotes fat deposition and increases backfat thickness (Cai et al., 2021). In this study, changes in the abundance of *Lactobacillus reuteri* and *Clostridium butyricum* in the 50W group were associated with reduced subcutaneous adipose tissue deposition and decreased backfat thickness in Diqing Tibetan pigs.

Based on transcriptomic sequencing data of subcutaneous adipose tissue, using thresholds of log_2_|FoldChange| > 1 and Padj < 0.05, 2,211 genes were identified as differentially expressed between the 0W and 50W groups. Genes associated with attenuated insulin signaling (*PYGB, RPS*6*KA*1, and *PIK*3*R*2) were significantly upregulated in the 50W group, while genes associated with glucose metabolism, adipogenesis, and lipid synthesis (*RPS*6*KB*1, *RBP*7, *B*4*GALT*6, *PCK*1, *GYS*2, *FFAR*4) were significantly downregulated (Figure 1C). Research indicates that the reduction of insulin signaling in adipose tissue activates lipolysis, leading to the breakdown of triglycerides into free fatty acids (FFAs) and reduced lipid content. These findings suggest that subcutaneous adipose tissue in the 50W group exhibited enhanced lipolysis and suppressed lipid synthesis.

Quantitative lipidomic analysis of subcutaneous adipose tissue, using thresholds of VIP > 1 and *P*-value < 0.05, detected 17 classes of metabolites (Supplementary Figure 1A), with 63 significantly altered lipid metabolites. Compared to the 0W group, triglyceride levels were significantly reduced, and free fatty acid levels were significantly elevated in the 50W group (Figure 1D). As free fatty acids are products of triglyceride hydrolysis, these results confirm active lipolysis in the 50W group.

Integrated multi-omics analysis revealed that *Clostridium butyricum* abundance was significantly negatively correlated with genes associated with attenuated insulin signaling (*PYGB, RPS*6*KA*1, *PIK*3*R*2) and positively correlated with *FFAR*4, a gene regulating adipogenesis and insulin sensitivity (Figure 1E). Transcriptome-lipidome correlation analysis showed that *PYGB, RPS*6*KA*1, and *PIK*3*R*2 were negatively correlated with triglyceride levels, while *FFAR*4 was positively correlated with triglyceride levels (Figure 1F). The Sankey diagram (Figure 1G) visually illustrates that changes in the abundance of *Clostridium butyricum* may influence lipid metabolism by regulating gene expression in adipose tissue, ultimately promoting triglyceride reduction and enhanced lipolysis.

In conclusion, replacing 50% soybean meal with walnut meal in the diet of Diqing Tibetan pigs was associated with a reduction in cecum *Clostridium butyricum* abundance and with changes in lipolytic activity in subcutaneous adipose tissue. This process decreased triglyceride content, increased free fatty acid levels, and ultimately reduced backfat thickness in Diqing Tibetan pigs.

### 3.4 Associations between cecum *Lactobacillus* abundance and hepatic fatty acid oxidation in Diqing Tibetan pigs

**Figure 2:**
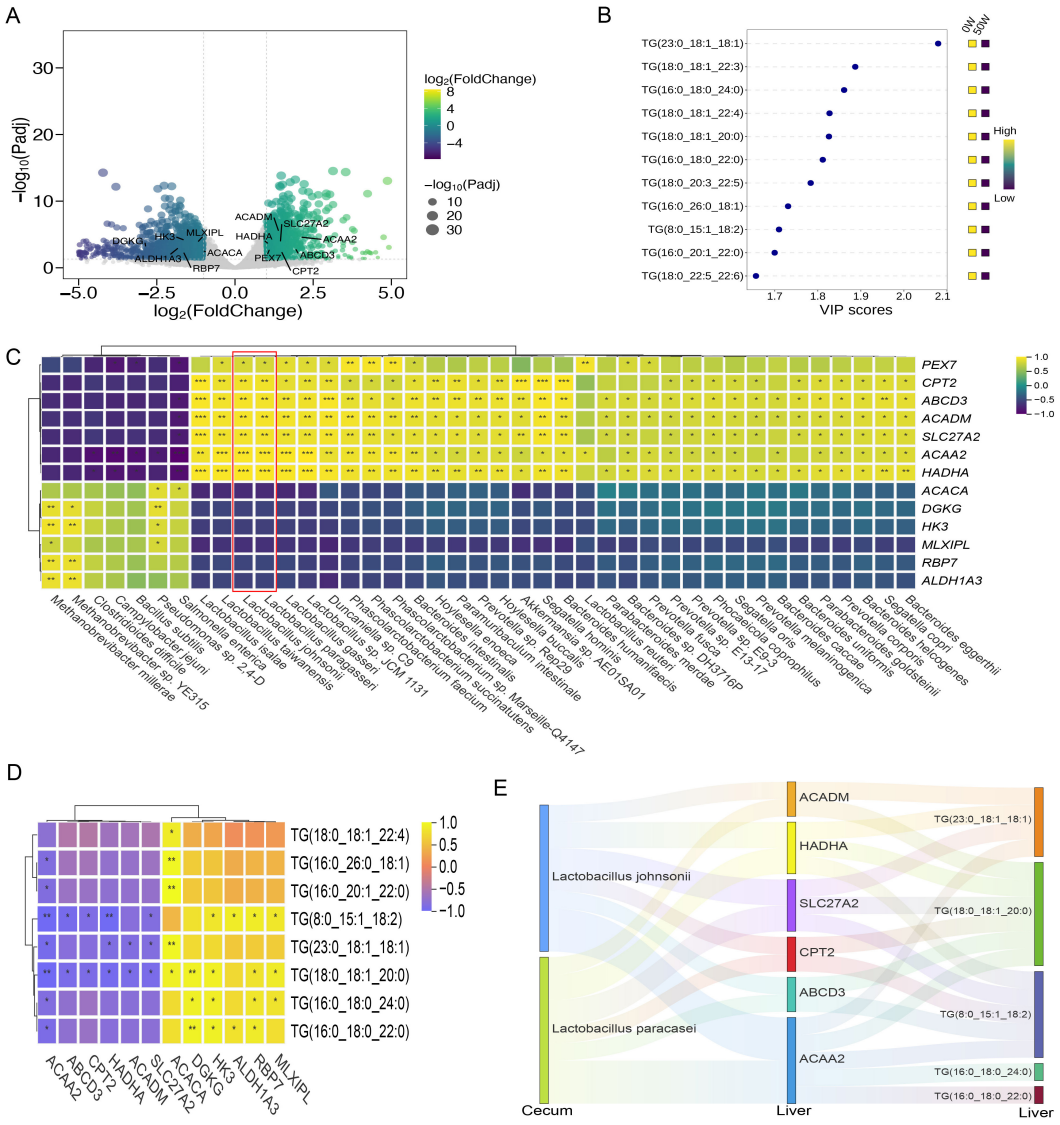
Integrated analysis of cecum metagenomics, hepatic transcriptomics, and lipidomics. **(A)** Transcriptomic analysis revealed differentially expressed genes in the liver tissue between the 0W and 50W groups. **(B)** Lipidomic analysis revealed differences in triglyceride metabolites in the liver tissue between the 0W and 50W groups. **(C)** Correlation analysis between cecum metagenomics and hepatic transcriptomics (Asterisks indicate the level of significance in correlation analysis: *P < 0.05, **P < 0.01). **(D)** Correlation analysis between hepatic transcriptomics and lipidomics (Asterisks indicate the level of significance in correlation analysis: *P < 0.05, **P < 0.01). **(E)** Sankey diagram showing how increased cecum Lactobacillus abundance may regulate hepatic genes to enhance fatty acid oxidation and reduce triglyceride accumulation.

Based on liver tissue transcriptomic sequencing data, using thresholds of log_2_|FoldChange| > 1 and Padj < 0.05, a total of 1,964 genes were identified as significantly differentially expressed in liver tissues. Functional annotation revealed that genes associated with triglyceride synthesis (*ACACA, MLXIPL, H*K3, *RBP*7, *ALDH*1*A*3, and *DGKG*) were significantly downregulated in the 50W group, while genes related to fatty acid β-oxidation (*PEX*7, *ACADM, HADHA, SLC*27*A*2, *CPT*2, *ABCD*3, and *ACAA*2) were significantly upregulated in the 50W group (Figure 2A). These results suggest that dietary substitution of 50% soybean meal with walnut meal is associated with altered hepatic lipid metabolism, including reduced expression of lipid synthesis-related genes and increased expression of fatty acid oxidation-related genes, which may contribute to decreased triglyceride accumulation in the liver.

Lipidomic analysis of liver tissue, using thresholds of VIP > 1 and *P*-value < 0.05, detected 27 lipid metabolite classes (Supplementary Figure 1B), with 125 significantly altered lipid metabolites. Among these, 11 triglyceride metabolites were significantly higher in the 0W group than in the 50W group (Figure 2B), consistent with transcriptomic observations of suppressed triglyceride synthesis and enhanced fatty acid oxidation.

Given the existence of the gut-liver axis, we conducted correlation analyses between cecum microbial abundance and hepatic differentially expressed genes. The results revealed a significant positive correlation between *Lactobacillus* abundance and the expression of genes regulating fatty acid oxidation in liver tissues (Figure 2C). Previous studies have confirmed that *Lactobacillus johnsonii* promotes fatty acid oxidation to reduce hepatic lipid deposition (Wang et al., 2017; Xu et al., 2023), while *Lactobacillus paracasei* prevents non-alcoholic fatty liver disease (NAFLD) by modulating gut microbiota and hepatic metabolism (Pan et al., 2022). Integrated analysis of liver transcriptomics and lipidomics further demonstrated that the expression of fatty acid oxidation-related genes was negatively correlated with triglyceride content, whereas genes involved in glucose metabolism and lipid synthesis showed a positive correlation with triglyceride levels (Figure 2D). In summary, replacing 50% of soybean meal with walnut meal increased the abundance of *Lactobacillus* in the cecum of Diqing Tibetan pigs. These findings indicate that increases in *Lactobacillus* abundance in the 50W group are associated with altered hepatic lipid metabolism, including enhanced fatty acid oxidation and reduced triglyceride accumulation. The Sankey diagram (Figure 2E) visually illustrates that increased cecal *Lactobacillus* abundance may promote hepatic fatty acid oxidation and reduce triglyceride accumulation by regulating the expression of fatty acid oxidation-related genes, thereby improving liver lipid metabolism.

In addition, carcass performance measurements showed that both fat content and backfat thickness were lower in the 50W group compared to the 0W group. As subcutaneous fat is a component of white adipose tissue (WAT), which primarily reduces fat deposition through triglyceride hydrolysis, the released free fatty acids (FFAs) can enter non-adipose tissues (e.g., liver and muscle) and be re-esterified into triglycerides for storage. Excessive FFA influx into the liver may drive triglyceride synthesis and contribute to fatty liver development (Liu et al., 2016; Vergani, 2019). Our findings suggest that walnut meal supplementation enhances lipolytic capacity in WAT while promoting intestinal proliferation of *Lactobacillus johnsonii* and *Lactobacillus paracasei*, which collectively may contribute to improved hepatic fatty acid oxidation and decreased triglyceride accumulation. These observations are consistent with a potential protective adaptive response of the liver to dietary changes.

### 3.5 Differences in gene expression and metabolite content in muscle

**Figure 3:**
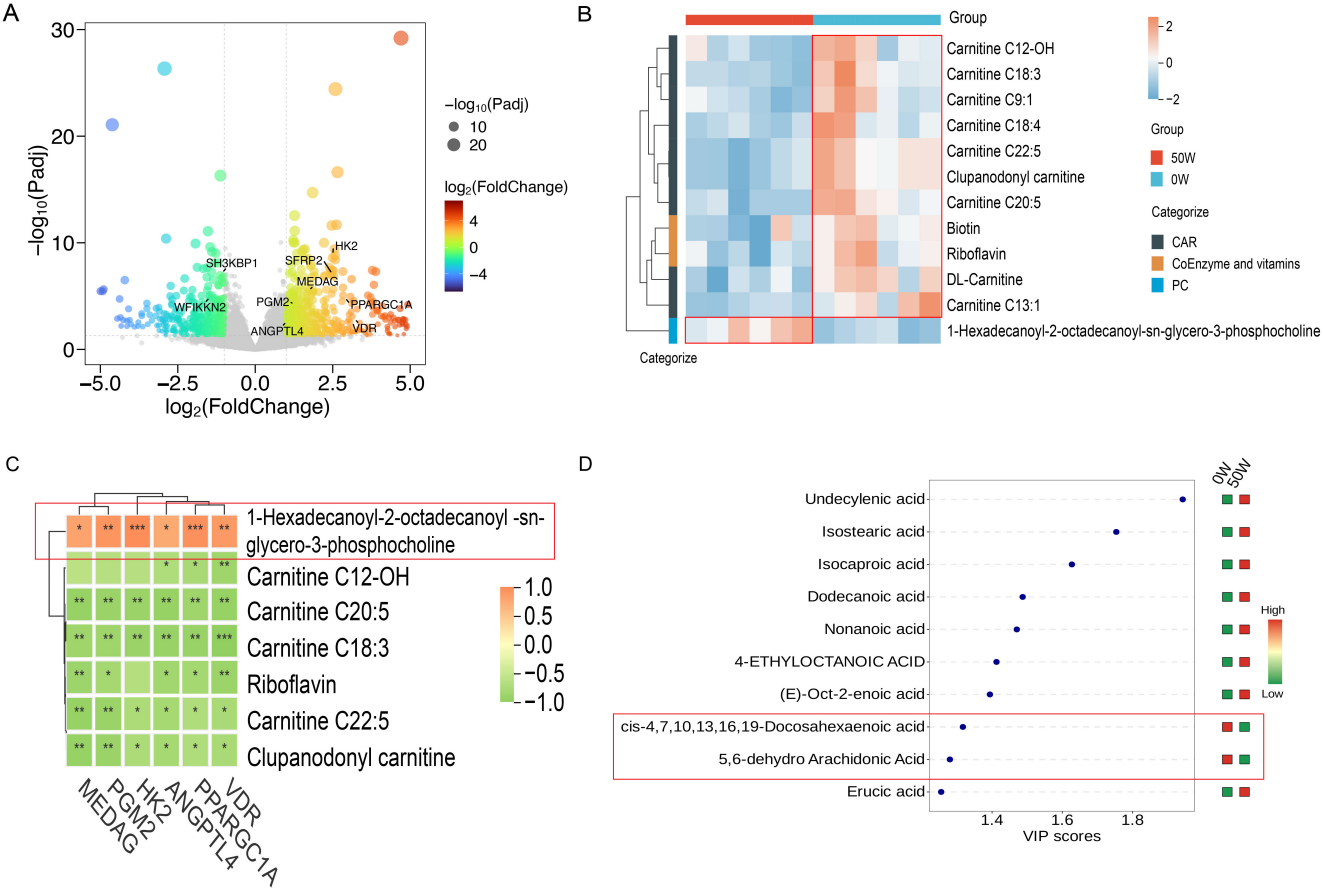
Integrated analysis of muscle transcriptomics and metabolomics. **(A)** Transcriptomic analysis revealed differentially expressed genes in the muscle tissue between the 0W and 50W groups. **(B)** Metabolomic analysis revealed differences in acylcarnitines, phosphatidylcholines, and coenzyme/vitamin metabolites in the muscle tissue between the 0W and 50W groups. **(C)** Correlation analysis between transcriptomics and metabolomics (Asterisks indicate the level of significance in correlation analysis: *P < 0.05, ** *P* < 0.01). **(D)** Metabolomic analysis revealed differences in serum free fatty acids between the 0W and 50W groups.

Based on transcriptomic sequencing data from the Longissimus thoracis muscle, using thresholds of log_2_|FoldChange| > 1 and Padj < 0.05, a total of 475 significantly differentially expressed genes were identified in muscle tissues. Genes associated with glucose metabolism (*HK*2 and *PGM*2), adipogenesis (*MEDAG*), and triglyceride synthesis (*PPARGC*1*A, VDR*, and *ANGPTL*4) were significantly upregulated in the 50W group, while genes related to muscle growth and development (*SH*3*KBP*1 and *WFIKKN*2) were significantly downregulated in the 50W group (Figure 3A). These results suggest that dietary walnut meal supplementation may promote increased utilization of glucose for triglyceride synthesis in the 50W group by upregulating lipid metabolism-related genes and downregulating muscle development-related genes.

Based on quantitative metabolite data from muscle tissues, using thresholds of VIP > 1 and *P*-value < 0.05, 33 distinct metabolite classes were detected between the 0W and 50W groups (Supplementary Figure 1C), with 111 significantly differential metabolites identified. Among these, acylcarnitine metabolites (9 types) were upregulated in the 0W group (Figure 3B). Studies indicate that acylcarnitines, as critical carriers in fatty acid oxidation, transport fatty acids into mitochondria for energy production (Reuter and Evans, 2012). Notably, the reduced levels of acylcarnitines in the 50W group may lower fatty acid oxidation capacity, diverting more fatty acids toward triglyceride synthesis. Among glycerophospholipid metabolites, phosphatidylcholine (1-palmitoyl-2-stearoyl-sn-glycero-3-phosphocholine) levels were significantly elevated in the 50W group (Figure 3B). Phosphatidylcholine plays a key role in lipid droplet formation and regulation, which serve as primary storage sites for triglycerides in adipocytes (Krahmer et al., 2011; Padilla-Benavides et al., 2016). Thus, increased phosphatidylcholine content in the 50W group may stabilize lipid droplets, facilitating triglyceride storage.

For coenzyme and vitamin metabolites, riboflavin and biotin levels were significantly reduced in the 50W group (Figure 3B). Riboflavin, through its metabolites FMN and FAD, acts as a coenzyme critical for fatty acid oxidation. Riboflavin deficiency impairs fatty acid oxidation capacity (Henriques et al., 2010; Zhu et al., 2014). In 3T3-L1 adipocytes, biotin supplementation enhances fatty acid oxidation while suppressing fatty acid synthesis (Moreno-Méndez et al., 2019). Therefore, decreased riboflavin and biotin levels in the 50W group may reduce fatty acid oxidation in muscle cells.

Integrated analysis of muscle transcriptomics and metabolomics revealed significant positive correlations between the expression of preadipocyte differentiation-related genes (*MEDAG*), triglyceride synthesis/storage-related genes (*ANGPTL*4, *PPARGC*1*A, VDR*), and glucose metabolism-related genes (*PGM*2, *HK*2) with phosphatidylcholine content, while showing significant negative correlations with acylcarnitine metabolite levels (Figure 3C). These findings suggest that the upregulated expression of adipogenic, lipid synthesis, and storage-related genes in the 50W group increases phosphatidylcholine content and reduces acylcarnitine levels in muscle tissues. Elevated phosphatidylcholine promotes lipid droplet formation, whereas decreased acylcarnitines diminish fatty acid oxidation capacity, redirecting fatty acids toward triglyceride synthesis and thereby increasing intramuscular fat content.

### 3.6 Ectopic fat deposition promotes intramuscular fat formation in Diqing Tibetan pigs

Alterations in gut microbiota influence adipose tissue function and metabolic status, highlighting the existence of a gut-adipose axis (Ejtahed et al., 2020; Kumari and Kozyrskyj, 2017). In this study, integrated analyses of cecum metagenomics, adipose tissue transcriptomics, and lipidomics revealed that substituting soybean meal with walnut meal reduced the abundance of *Clostridium butyricum* in the cecum of Diqing Tibetan pigs. This reduction downregulated adipocyte differentiation-related genes and may contribute to attenuated insulin signaling in back subcutaneous adipose tissue. Reduced insulin signaling in adipose tissue enhances lipolysis, promoting triglyceride hydrolysis and reducing adipose triglyceride content, ultimately decreasing backfat thickness in Diqing Tibetan pigs.

Subcutaneous adipose tissue is the primary fat storage depot in pigs (Kouba et al., 1999). When insulin signaling in adipose tissue is attenuated, excessive free fatty acids (FFAs) are released into circulation, which are subsequently transported to and stored in non-adipose organs—a process termed ectopic fat deposition (Snel et al., 2012). In serum from 0W and 50W groups of Tibetan pigs, we identified 10 FFAs with significant differences. In the serum of Tibetan pigs from the 0W and 50W groups, we identified 10 free fatty acids with significantly different levels (Figure 3D). Among these, 2 polyunsaturated fatty acids—DHA (cis-4,7,10,13,16,19-Docosahexaenoic acid) and a 5,6-dehydro arachidonic acid derivative—were significantly higher in the 0W group compared to the 50W group. In contrast, the remaining 8 free fatty acids exhibited significantly elevated levels in the 50W group relative to the 0W group (Figure 3D). Studies indicate that DHA supplementation enhances tissue glucose utilization and insulin sensitivity (Capel et al., 2015; Flachs et al., 2006), thereby promoting lipid synthesis in insulin-sensitive adipose tissue. However, excessive FFAs impair glucose transport and utilization in adipocytes, reducing insulin responsiveness in adipose tissue (Boden, 2011). Notably, arachidonic acid uniquely enhances glucose uptake and insulin signaling in adipocytes (Nugent et al., 2001). The reduced DHA and arachidonic acid levels in the 50W group may contribute to attenuated adipose tissue insulin signaling, driving lipolysis and FFA release into circulation. Elevated FFAs provide substrates for intramuscular triglyceride synthesis via esterification.

In this study, increased *Lactobacillus* abundance in the gut upregulated hepatic fatty acid oxidation genes, reducing liver lipid deposition. Concurrently, back subcutaneous adipose tissue in the 50W group exhibited attenuated insulin signaling, while the Longissimus thoracis muscle showed significant upregulation of genes promoting preadipocyte differentiation (*MEDAG* and *VDR*). *MEDAG* drives preadipocyte differentiation into mature adipocytes (Zhang et al., 2012), and *VDR* enhances adipogenesis and triglyceride accumulation (Nimitphong et al., 2012). The expression of these genes in muscle facilitates preadipocyte differentiation. During differentiation, newly formed adipocytes esterify circulating FFAs into triglycerides for storage, increasing intramuscular triglyceride content and promoting ectopic intramuscular fat deposition.

### 3.7 Molecular regulatory mechanisms underlying walnut meal-induced intramuscular fat deposition in Diqing Tibetan pigs

**Figure 4:**
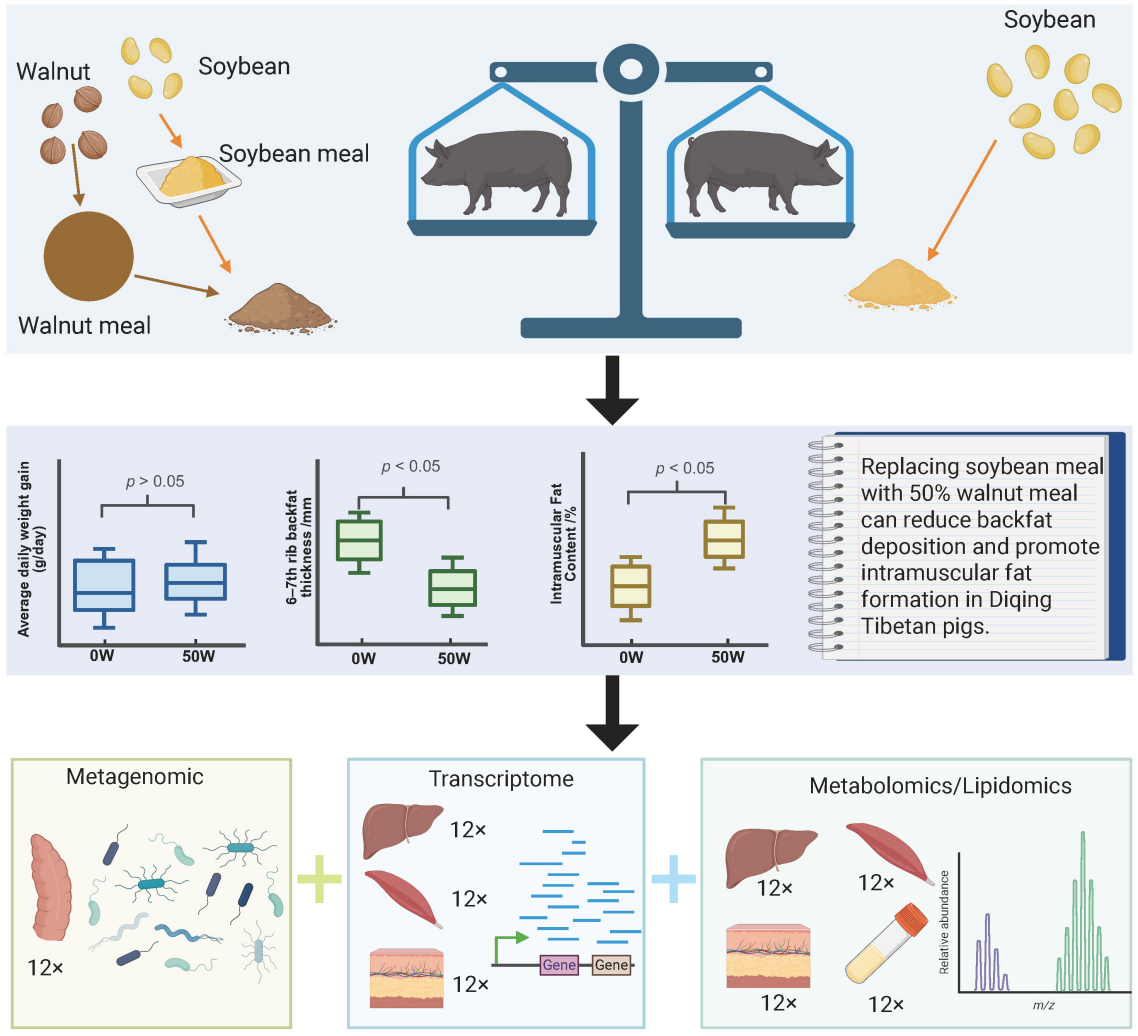
Replacing 50% of soybean meal with walnut meal can significantly reduce subcutaneous fat and increase intramuscular fat content in Diqing Tibetan pigs without affecting growth performance (the figure was created using BioRender).

In this study, we conducted a feeding trial in fattening Diqing Tibetan pigs using graded levels of walnut meal to replace soybean meal (25%, 50%, and 100%). We found that a 50% replacement not only did not compromise fattening performance but also significantly reduced dorsal subcutaneous fat deposition and promoted intramuscular fat accumulation. Based on these results, we collected cecum contents for metagenomic sequencing, and performed transcriptomic analysis of the liver, muscle, and dorsal subcutaneous fat. In addition, lipidomic profiling was conducted on the liver and subcutaneous fat, while metabolomic profiling was performed on muscle and serum (Figure 4).

**Figure 5:**
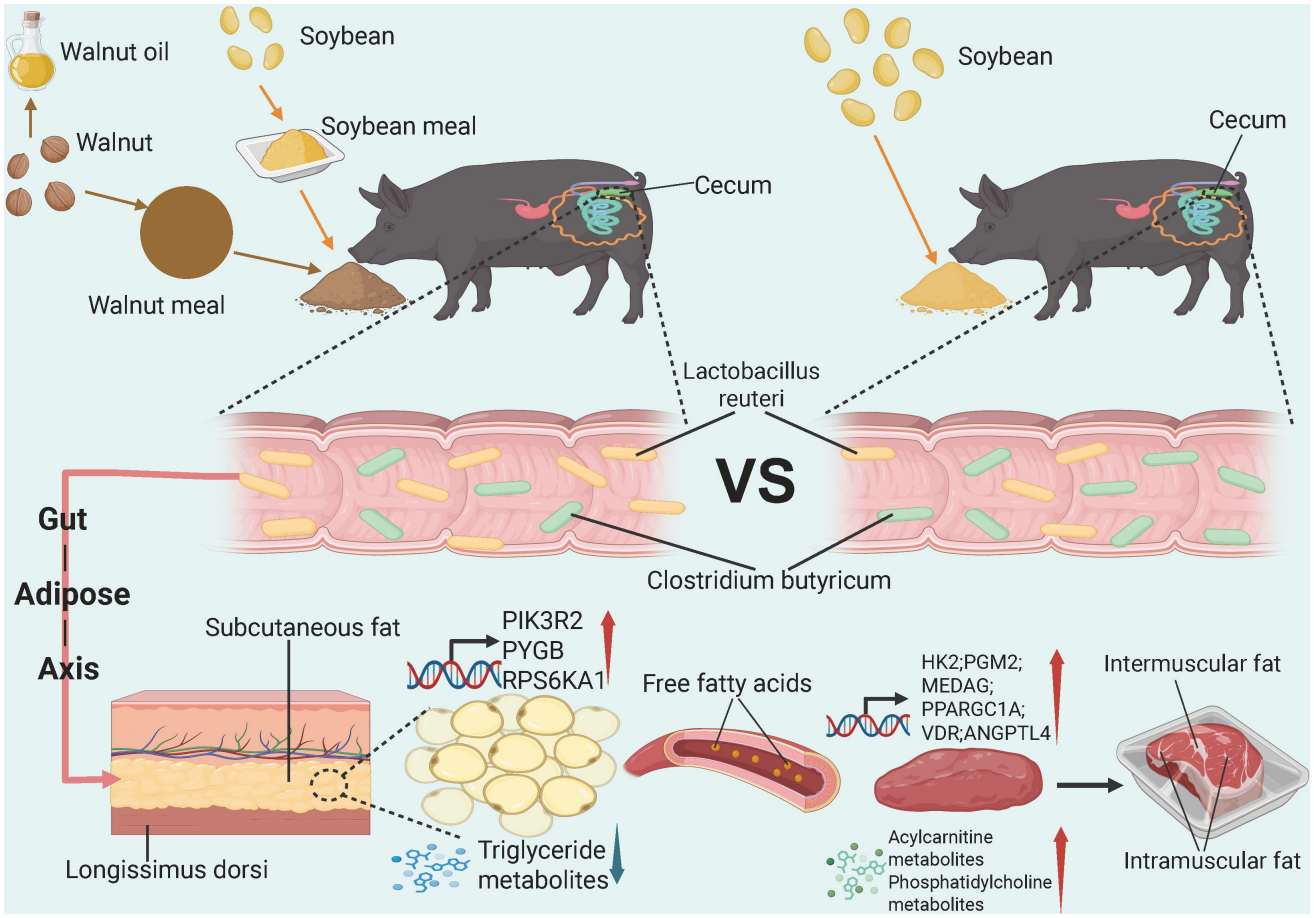
Molecular regulatory network of walnut meal replacing soybean meal-induced intramuscular fat accumulation in Diqing Tibetan pigs (the figure was created using BioRender).

Integrated multi-omics analysis revealed that replacing 50% of soybean meal with walnut meal in the diet of Diqing Tibetan pigs altered the abundance of *Clostridium* species in the gut. Specifically, the reduction of *Clostridium butyricum* may weaken the insulin signaling pathway-related genes (*PIK*3*R*2, *PYGB*, and *RPS*6*KA*1) in subcutaneous adipose tissue through the gut-adipose axis. This process leads to a decrease in triglyceride content and an increase in free fatty acid levels in adipose tissue. Adipose tissue with enhanced lipolytic activity releases free fatty acids into the bloodstream, which are then transported to muscle tissue via the circulation. In the muscle, the upregulation of glucose metabolism-related genes (*HK*2 and *PGM*2), adipocyte differentiation-related genes (*MEDAG*), and triglyceride synthesis-related genes (*PPARGC*1*A, VDR*, and *ANGPTL*4) promotes adipocyte formation and triglyceride synthesis. Meanwhile, the increase in phosphatidylcholine levels and decrease in acylcarnitine levels favor the formation of lipid droplets in adipocytes while inhibiting the oxidation of free fatty acids in muscle tissue. These metabolic changes result in muscle adipocytes absorbing free fatty acids from the blood and storing them in the form of triglycerides through esterification. Ultimately, this promotes the accumulation of triglycerides in muscle tissue, thereby increasing the content of intramuscular and intermuscular fat (Figure 5).

## 4 Discussion

In the process of lipid synthesis and storage in adipose tissue, preadipocytes play a crucial role. Preadipocytes can differentiate into mature adipocytes, which convert free fatty acids into triglycerides via esterification for storage. Therefore, promoting adipogenesis enhances the lipid storage capacity of adipose tissue, reduces the overflow of free fatty acids into the bloodstream, and improves insulin resistance, representing a potential therapeutic strategy for metabolic diseases (Chitraju et al., 2017; Sánchez-Ceinos et al., 2021). Transcriptomic analysis of back subcutaneous adipose tissue in this study revealed significantly lower expression of genes associated with adipogenesis and lipid synthesis in the 50W group compared to the 0W group. Metabolomic analysis further confirmed a marked decrease in triglyceride content and a significant increase in free fatty acids (FFAs) in the 50W group. These findings suggest enhanced lipolysis in the back subcutaneous adipose tissue of the 50W group. Serum metabolomics also showed elevated FFA levels in the 50W group, indicating that lipolysis-derived FFAs were released into circulation, increasing the risk of ectopic lipid deposition.

As a major lipid storage depot in pigs, back subcutaneous adipose tissue under insulin-resistant conditions releases FFAs into the bloodstream, which are subsequently deposited in non-adipose tissues such as the liver and skeletal muscle (ectopic lipid deposition). The liver, as the central regulator of lipid metabolism, becomes prone to non-alcoholic fatty liver disease (NAFLD) when hepatic metabolic functions are impaired during insulin resistance—including the transport of triglycerides via very-low-density lipoprotein (VLDL) to adipose tissue for storage (Heeren and Scheja, 2021) or fatty acid β-oxidation (Nie et al., 2024). This study observed that increased abundance of *Lactobacillus* in the cecum enhanced hepatic oxidative metabolism, potentially mitigating the risk of hepatic lipid accumulation caused by active lipolysis in white adipose tissue. Concurrently, elevated circulating FFAs may promote intramuscular lipid synthesis, increasing intramuscular fat content.

Beyond the liver, skeletal muscle is another major site of ectopic lipid deposition. Girousse et al. demonstrated that the release of preadipocytes from back subcutaneous adipose tissue contributes significantly to ectopic adipocyte deposition, particularly in skeletal muscle (Girousse et al., 2019). Upon differentiation into mature adipocytes within muscle tissue, these cells absorb and utilize FFAs for triglyceride synthesis and storage. Ectopic lipid deposition in skeletal muscle is a key biological process for enhancing intramuscular fat content in pork.

This study revealed significantly upregulated expression of key adipogenic genes (*MEDAG* and *VDR*) in the Longissimus thoracis muscle of the 50W group. Although myocytes possess the capacity to absorb and oxidize FFAs for energy, metabolomic analysis showed reduced levels of acylcarnitines alongside increased biotin and coenzyme-related metabolites in the 50W group, suggesting suppressed fatty acid oxidation. FFAs released from back subcutaneous adipose tissue may instead be utilized by intramuscular adipocytes for triglyceride synthesis, ultimately leading to significantly higher intramuscular fat content in the 50W group.

This study observed that replacing soybean meal with walnut meal significantly increased the abundance of *Lactobacillus reuteri* in the cecum while decreasing the abundance of *Clostridium butyricum*. The reduction of *Clostridium butyricum* is likely attributable to the proliferation of *Lactobacillus reuteri*. Members of the genus *Lactobacillus* produce lactic acid through fermentation, thereby lowering intestinal pH. Since *Clostridium* species are generally sensitive to acidic environments, this lower pH creates unfavorable conditions for their growth (Guo et al., 2017; Naaber et al., 2004; Jiao et al., 2022). Therefore, a walnut meal diet likely influences the gut microbiota by promoting an acidic environment that favors *Lactobacillus* while inhibiting *Clostridium*.

## 5 Conclusion

This study investigated the molecular mechanisms by which replacing 50% soybean meal with walnut meal in a corn-soybean meal-based diet enhances intramuscular fat content in finishing-phase Diqing Tibetan pigs, using integrated multi-omics approaches (metagenomics, transcriptomics, and lipidomics). The results showed that replacing 50% soybean meal with walnut meal significantly reduced the abundance of *Clostridium butyricum* in the cecum. *Clostridium butyricum* promoted the lipolysis of white adipose tissue, increasing the release of free fatty acids (FFAs) into the bloodstream. Meanwhile, skeletal muscle tissue inhibited fatty acid oxidation, promoting adipocyte differentiation and triglyceride synthesis, which converted FFAs into stored triglycerides. These processes ultimately led to an increase in intramuscular fat content in the muscle tissue. The findings provide a scientific basis for the development and utilization of regional unconventional feed resources and offer a feasible strategy for the production of high-quality marbled pork.

## Data Availability

The data presented in this study are publicly available. The metagenomic and transcriptomic data have been deposited in the China National Center for Bioinformation (CNCB) under the following accession numbers: CRA025597 (metagenomic) and CRA026468 (transcriptomic). The metabolomic data have been uploaded to the MetaboLights database under the following accession numbers: MTBLS12571 (serum), MTBLS12579 (muscle), MTBLS12580 (fat), and MTBLS12583 (liver).
