# Supplementary material for: Feeding Diqing Tibetan pigs with 50% of soybean meal replaced by walnut meal can reduce subcutaneous fat deposition and promote intramuscular fat accumulation
Source: Front Microbiol. 2026 Mar 19;17:1794046. doi: 10.3389/fmicb.2026.1794046 (PMC13044152; doi:10.3389/fmicb.2026.1794046)
Supplement: Supplementary file 1 [file Supplementary_file_1.docx]

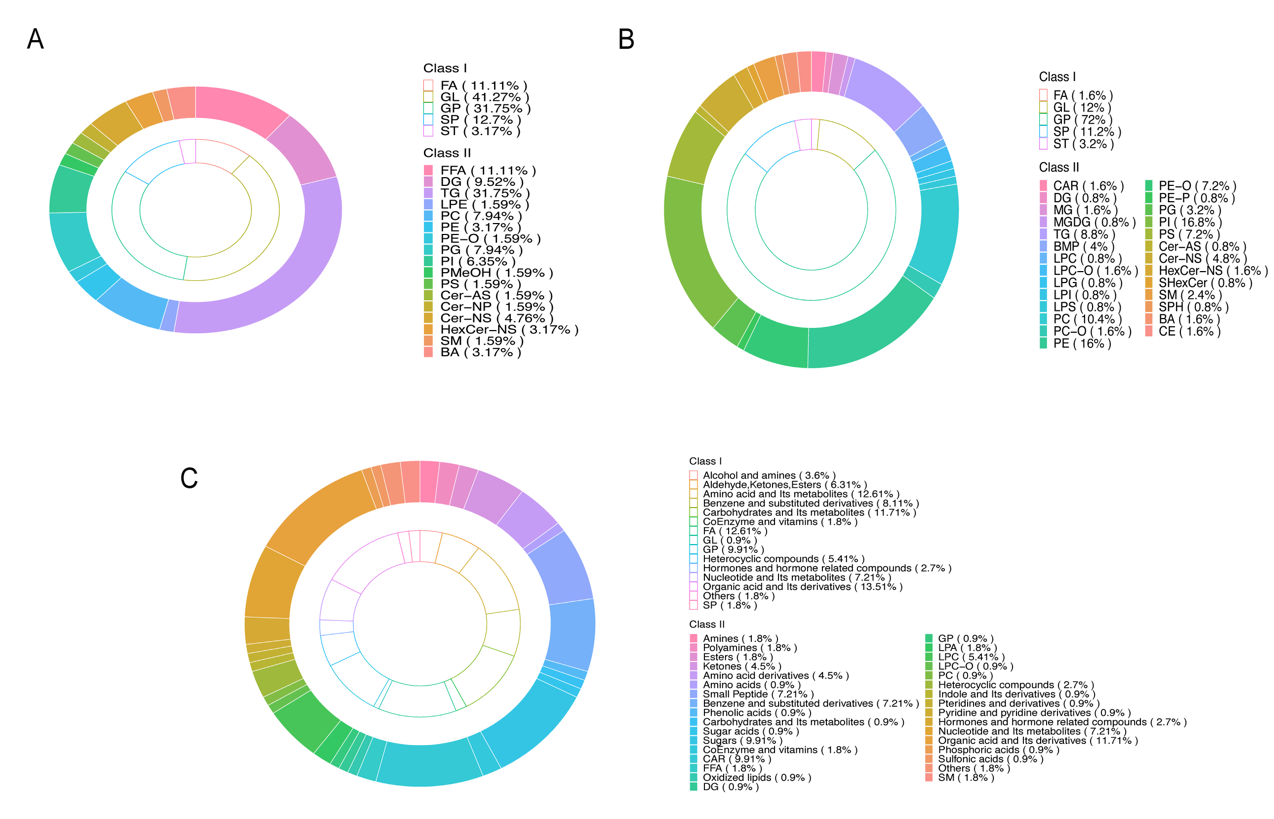


**Figure S1 The types of metabolites detected in adipose, liver, and muscle tissues. A** The metabolites and their relative proportions detected in adipose tissue in the 0W and 50W groups. **FA**-Fatty acyls, **GL**-Glycerolipids, **GP**-Glycerophospholipids, **SP**-Sphingolipids, **ST**-Sterol lipids. **BA**-Bile acids, **CAR**-Acylcarnitines, **Cer-AP**-Ceramide, **Cer-AS**-Ceramide, **Cer-NP**-Ceramide, **Cer-NS**-Ceramide, **Cholesterol** -Cholesterol, **DG**-Diglyceride, **DG-O**-Diglyceride (ether-linked), **DGDG**-Digalactosyldiglyceride, **Eicosanoid**-Oxidized lipid, **FFA**-Free fatty acid, **HexCer-AP**-Glycosphingolipid, **HexCer-NS**-Glycosphingolipid, **LNAPE**-N-Acyl-lysophosphatidylethanolamine, **LPC**-Lysophosphatidylcholine, **LPC-O**-Lysophosphatidylcholine (ether-linked), **LPE**-Lysophosphatidylethanolamine, **LPE-P**-Lysophosphatidylethanolamine (plasmalogen), **LPG**-Lysophosphatidylglycerol, **LPI**-Lysophosphatidylinositol, **LPS**-Lysophosphatidylserine, **MG**-Monoglyceride, **PA**-Phosphatidic acid, **PC**-Phosphatidylcholine, **PC-O**-Phosphatidylcholine (ether-linked), **PE**-Phosphatidylethanolamine, **PE-O**- Phosphatidylethanolamine (ether-linked), **PE-P**-Phosphatidylethanolamine (plasmalogen), **PG**- Phosphatidylglycerol, **PI**-Phosphatidylinositol**, PMeOH**-Phosphatidylmethanol, **PS**-Phosphatidylserine, **SM**-Sphingomyelin, **SPH**-Sphingosine, **TG**-Triglyceride, **TG-O**-Triglyceride (ether-linked)

**B** The metabolites and their relative proportions detected in liver tissue in the 0W and 50W groups. **BA**-Bile acids, **CAR**-Acylcarnitines, **Cer-AP**-Ceramide, **Cer-AS**-Ceramide, **Cer-NP**-Ceramide, **Cer-NS**-Ceramide, **HexCer-AP**-Hexosylceramide, **HexCer-NS**-Hexosylceramide, **DG**-Diacylglycerol, **DG-O**-Ether-linked diacylglycerol, **DGDG**-Digalactosyldiacylglycerol, **MG**- Monoacylglycerol, **TG**-Triacylglycerol, **TG-O**-Ether-linked triacylglycerol, **FFA**-Free fatty acids, **Eicosanoid**-Oxidized lipids, **LNAPE**-N-acyl lysophosphatidylethanolamine, **LPC**-Lysophosphatidylcholine, **LPC-O**-Ether-linked lysophosphatidylcholine, **LPE**-Lysophosphatidylethanolamine, **LPE-P**-Plasmalogen lysophosphatidylethanolamine, **LPG** Lysophosphatidylglycerol, **LPI**-Lysophosphatidylinositol, **LPS**-Lysophosphatidylserine, **PA**-Phosphatidic acid, **PC**-Phosphatidylcholine, **PC-O**-Ether-linked phosphatidylcholine, **PE**-Phosphatidylethanolamine, **PE-O**-Ether-linked phosphatidylethanolamine, **PE-P**-Plasmalogen phosphatidylethanolamine, **PG**-Phosphatidylglycerol, **PI**-Phosphatidylinositol, **PMeOH**-Phosphatidylmethanol, **PS**-Phosphatidylserine, **SM**-Sphingomyelin, **SPH**-Sphingosine.

**C** The metabolites and their relative proportions detected in muscle tissue in the 0W and 50W groups.

**FA-**Fatty Acyls**, GP-**Glycerophospholipids, **SP-**Sphingolipids**, GL-**Glycerolipids. **PA**- Phosphatidic acid, **PC**-Phosphatidylcholine, **PC-O**-Alkylphosphatidylcholine, **PE**- Phosphatidylethanolamine, **PE-P**-Plasmenylethanolamine, **PG**-Phosphatidylglycerol, **PI**-Phosphatidylinositol, **LPA**-Lysophosphatidic acid, **LPC**-Lysophosphatidylcholine, **LPC-O**-Alkyl-lysophosphatidylcholine, **LPE**-Lysophosphatidylethanolamine, **LPE-P**-Plasmenyl-lysophosphatidylethanolamine, **LPS**-Lysophosphatidylserine, **MG**-Monoglyceride, **DG**-Diglyceride, **FFA**-Free fatty acid, **Cer**-Ceramide, **SPH**-Sphingosine, **SM**-Sphingomyelin, **CAR**-Acylcarnitine.
